# Association between physical activity and blood pressure control among Moroccan adults with hypertension

**DOI:** 10.3389/fspor.2026.1896600

**Published:** 2026-09-03

**Authors:** Chaimaa Chourite, Hanae Khossossi, Mustapha Mouilly

**Affiliations:** Interdisciplinary Laboratory of Sport Sciences, Institute of Sport Profession, Ibn Tofail University, Kenitra, Morocco

**Keywords:** blood pressure control, hypertension, lifestyle factors, Morocco, physical activity

## Abstract

**Background:**

Hypertension remains one of the leading causes of cardiovascular morbidity and premature mortality worldwide, with blood pressure control rates remaining particularly in low- and middle-income countries. In Morocco, the relationship between physical activity levels and blood pressure control among hypertensive adults remains insufficiently documented. Accordingly, this study aimed to estimate the prevalence of uncontrolled blood pressure and determine whether graded physical activity levels were independently associated with blood pressure control among treated hypertensive adults attending Moroccan primary healthcare center.

**Methods:**

A cross-sectional study included 612 treated hypertensive adults consecutively recruited during routine follow-up at a primary healthcare center in Kenitra, Morocco, between July and December 2025. Physical activity was assessed using the validated Moroccan IPAQ. Blood pressure was measured, and was interpreted according to the 2023 European Society of Hypertension recommendations. Multivariable logistic regression adjusted for age, sex, income, family history, smoking, salt intake, waist-to-height ratio, diabetes and dyslipidemia, medication adherence, and antihypertensive treatment. Candidate variables were selected at *p* ≤ 0.20, multicollinearity was assessed using variance inflation factors, and model calibration was evaluated using the Hosmer–Lemeshow test.

**Results:**

The mean age of participants was 58.5 ± 11.1 years, and 57.0% were women. Of the 612 participants, 36.1% had low physical activity, 39.1% had moderate physical activity, and 24.8% had high physical activity. Overall, 66.0% had uncontrolled blood pressure. Compared with low physical activity, moderate physical activity was associated with 51% lower odds of uncontrolled blood pressure (AOR = 0.49; 95% CI: 0.31–0.77), whereas high physical activity was associated with 69% lower odds (AOR = 0.31; 95% CI: 0.18–0.52). Higher odds of uncontrolled blood pressure were observed among participants aged >60 years, current smokers, those with an elevated waist-to-height ratio or high salt intake, and those with combined diabetes and dyslipidemia.

**Conclusion:**

Greater physical activity was linked to more favorable blood pressure control among treated hypertensive adults in Morocco. These findings highlight the potential relevance of physical activity to improve cardiovascular outcomes in Morocco.

## 1 Introduction

Despite major advances in diagnosis and treatment, hypertension remains one of the leading global causes of cardiovascular morbidity, disability, and premature mortality (1, 2). More than one billion adults worldwide are currently living with hypertension, and approximately three-quarters reside in low- and middle-income countries (LMICs), where health systems often face limited resources for long-term chronic disease management (3, 4). The burden of uncontrolled blood pressure is particularly concerning in LMICs and Sub-Saharan African settings, where many treated hypertensive patients fail to achieve recommended blood pressure targets (5). This persistent gap exposes patients to preventable complications, including stroke, heart failure, chronic kidney disease, and premature death.

Although pharmacological treatment is effective and widely recommended, medication alone is often insufficient to achieve optimal blood pressure control (6). Poor adherence, therapeutic inertia, limited follow-up, treatment costs, and insufficient integration of lifestyle counseling may reduce the effectiveness of pharmacological strategies in routine care (6, 7). Lifestyle determinants play a central role in both the development and control of hypertension. Physical inactivity, excess adiposity, high salt intake, smoking, and unhealthy dietary patterns are well-established contributors to poor blood pressure control and increased cardiovascular risk (8).

Among these modifiable factors, physical activity is a cornerstone of hypertension prevention and management. Evidence from randomized trials and meta-analyses has shown that regular aerobic, resistance, and combined exercise can produce clinically meaningful reductions in systolic and diastolic blood pressure (9–13). These benefits may be explained by improved endothelial function, reduced sympathetic nervous system activity, enhanced insulin sensitivity, improved vascular compliance, and favorable effects on body composition (14, 15).

In line with the WHO PEN-Plus strategy, strengthening hypertension care in LMICs requires moving beyond pharmacological treatment alone toward integrated, long-term, and community-oriented management. Identifying modifiable lifestyle determinants, particularly physical activity, is therefore essential for developing low-cost and scalable interventions in primary healthcare settings (16, 17). However, evidence from Morocco remains limited. Few studies have specifically examined the Although physical activity is recognized as an important component of hypertension management, its relationship with blood pressure control among treated hypertensive adults remains insufficiently characterized in Morocco. Previous national studies have largely focused on hypertension prevalence, general cardiovascular risk factors, or uncontrolled hypertension without specifically examining graded physical activity levels in relation to standardized blood pressure control within primary healthcare settings. In addition, limited evidence has assessed whether this association persists after accounting for relevant sociodemographic, behavioral, anthropometric, cardiometabolic, and treatment-related factors. Accordingly, this study aimed to estimate the prevalence of uncontrolled blood pressure and examine the independent association between physical activity levels and blood pressure control among treated hypertensive adults attending primary healthcare centers in Kenitra, Morocco.

## 2 Materials and methods

### 2.1 Study design and population

A cross-sectional study was conducted between July and December 2025 in public primary healthcare center in Kenitra, Morocco. This center provides routine follow-up and treatment servicesfor patients with hypertension. Eligible patients attending the participating center during the study period were screened and recruited consecutively during their routine hypertension follow-up consultations.

Eligible participants were adults aged ≥18 years with a confirmed diagnosis of hypertension and receiving antihypertensive treatment. The exclusion criteria were pregnancy, a history of major cardiovascular events, severe mobility-limiting disability, and acute illness at the time of assessment. All participants provided written informed consent before data collection.

The minimum sample size was estimated using Cochran's formula for a single population proportion, assuming a 95% confidence level, an expected proportion of 50% to provide the most conservative estimate, and a 5% margin of error. This calculation yielded a minimum required sample of 385 participants. Recruitment continued throughout the predefined study period resulting in a final analytical sample of 612 participants.

The study was conducted in accordance with the Declaration of Helsinki. The study protocol was approved by the Ethics Committee of the Faculty of Medicine and Pharmacy, Mohammed V University of Rabat.

### 2.2 Data collection tools

#### 2.2.1 Demographic and clinical information

Data were collected using a structured interviewer-administered questionnaire. Information obtained included sociodemographic characteristics, personal and family history of hypertension, treatment-related variables, and lifestyle behaviors.

#### 2.2.2 Physical activity assessment

Physical activity was assessed using the interviewer-administered International Physical Activity Questionnaire (IPAQ). Participants reported the frequency, expressed as days per week, and duration, expressed as minutes per day, of walking and moderate- and vigorous-intensity physical activities performed during the preceding seven days. Daily sitting time was also recorded as an indicator of sedentary behavior.

Physical activity volume was calculated in metabolic equivalent minutes per week (MET-min/week) by multiplying the reported duration and frequency of each activity by the corresponding IPAQ metabolic equivalent value: 3.3 METs for walking, 4.0 METs for moderate-intensity activity, and 8.0 METs for vigorous-intensity activity. Total physical activity was obtained by summing the walking, moderate-intensity, and vigorous-intensity MET-min/week scores. Participants were classified as having low (<600 MET-min/week), moderate (600–2,999 MET-min/week), or high (≥3,000 MET-min/week) physical activity levels according to the standard IPAQ scoring protocol.

#### 2.2.3 Blood pressure measurement and control

Blood pressure was measured using a validated automated oscillometric device with an appropriately sized cuff after at least five minutes of seated rest. Two measurements were obtained at one-minute intervals, and the mean value was used for analysis. Blood pressure control was defined according to the 2023 European Society of Hypertension guidelines (18). Using age-specific office blood pressure targets for treated hypertensive adults.

#### 2.2.4 Covariates

Lifestyle covariates included smoking status (current smoker/non-smoker), alcohol consumption (yes/no), and salt consumption practice (hight/low), based on self-report. Salt intake was estimated from habitual discretionary salt use and frequent consumption of salty foods. Participants reporting regular table salt use and/or frequent intake of high-sodium foods were classified as having high salt intake. Treatment-related variables included number of antihypertensive medications (monotherapy vs. ≥ 2 drugs), use of major antihypertensive classes, and self-reported medication adherence categorized as good, fair, or poor. These variables were selected *a priori* based on their documented associations with blood pressure control.

### 2.3 Statistical analysis

Data were entered into Microsoft Excel and analyzed using R software. Continuous variables were summarized as means ± standard deviations, whereas categorical variables were presented as frequencies and percentages. Comparisons across physical activity categories were performed using analysis of variance for continuous variables and Pearson's chi-square test for categorical variables. Variables with *p* ≤ 0.20 in univariable analyses were considered for inclusion in the multivariable logistic regression model. This relatively inclusive threshold was selected to reduce the risk of prematurely excluding potentially important confounders that might not demonstrate strong unadjusted associations. Variables considered clinically or epidemiologically relevant were also retained where appropriate, irrespective of their univariable *p*-values. Multicollinearity was assessed using variance inflation factors (VIF), with values ≥5 considered indicative of potentially problematic collinearity; the maximum observed VIF was estimated at 3.41. Model calibration was evaluated using the Hosmer–Lemeshow goodness-of-fit test, which provisionally indicated adequate fit (*χ*^2^ = 7.12, *p* = 0.524). Associations were reported as adjusted odds ratios (AORs) with 95% confidence intervals. Statistical significance was set at *p* < 0.05.

## 3 Results

### 3.1 Characteristics of participants with hypertension

A total of 612 adults with hypertension were included, of whom 349 (57.0%) were women. The mean age was 58.5 ± 11.1 years. Regarding physical activity, 221 participants (36.1%) had low physical activity, 239 (39.1%) had moderate physical activity, and 152 (24.8%) had high physical activity. Age did not differ significantly across physical activity categories (*p* = 0.800). Women accounted for 58.8% of the low physical activity group, 42.7% of the moderate physical activity group, and 77.0% of the high physical activity group. Educational attainment differed significantly across physical activity categories (*p* = 0.020), whereas marital status and monthly income showed no significant differences. The distribution of cardiometabolic comorbidities was comparable across groups (*p* = 0.180). Elevated waist-to-height ratio was highly prevalent overall (82.7%) and did not differ significantly across physical activity categories (*p* = 0.582). Similarly, body mass index categories were comparable across groups (*p* = 0.433), whereas smoking status differed significantly according to physical activity level (*p* = 0.022) (Table 1).

**Table 1:** Baseline characteristics of participants by physical activity (*N* = 612).

Characteristics	All participants (*n* = 612)	Low PA (*n* = 221)	Moderate PA (*n* = 239)	High PA (*n* = 152)	*p*-value
Gender, Female *n* (%)	349 (57.0)	130 (58.8)	102 (42.7)	117 (77.0)	0.038
Age (years)	58.5 ± 11.07	59 ± 11	58 ± 11	58 ± 10	0.800
Education level, *n* (%)					
Illiterate	424 (69.3)	114 (51.6)	141 (59.0)	139 (91.4)	0.020
Primary school	129 (21.1)	35 (15.8)	46 (19.2)	48 (31.6)	
Secondary school	35 (5.7)	10 (4.5)	14 (5.9)	11 (7.2)	
High school or more	24 (3.9)	16 (7.2)	4 (1.7)	4 (2.6)	
Marital status, *n* (%)					
Married	389 (63.6)	134 (60.6)	125 (52.3)	130 (85.5)	0.627
Not married	223 (36.4)	71 (32.1)	80 (33.5)	72 (47.4)	
Monthly income, *n* (%)					
Low	305 (49.8)	103 (46.6)	99 (41.4)	103 (67.8)	0.350
Middle	253 (41.3)	77 (34.8)	97 (40.6)	79 (52.0)	
High	54 (8.8)	25 (11.3)	9 (3.8)	20 (13.2)	
Family history of hypertension, *n* (%)					
Yes	363 (59.3)	139 (62.9)	112 (46.9)	112 (73.7)	0.010
No	249 (40.7)	66 (29.9)	93 (38.9)	90 (59.2)	
Comorbidities, *n* (%)					
Without diabetes or dyslipidemia	325 (53.1)	106 (46.0)	110 (71.7)	109 (71.7)	0.180
Diabetes mellitus alone	104 (17.0)	26 (11.8)	43 (18.0)	35 (23.0)	
Dyslipidemia alone	110 (18.0)	45 (20.4)	33 (13.8)	32 (23.1)	
Diabetes and dyslipidemia	73 (11.9)	28 (12.7)	19 (7.9)	26 (17.1)	
WHtR					
Normal	106 (17.3)	37 (18.0)	31 (15.1)	38 (18.8)	0.582
Elevated	506 (82.7)	168 (82.0)	174 (84.9)	164 (81.2)	
BMI (kg/m^2^)					
Normal	170 (27.8)	47 (22.9)	62 (30.2)	61 (30.2)	0.433
Overweight	241 (39.4)	84 (41.0)	79 (38.5)	78 (38.6)	
Obesity	201 (32.8)	74 (36.1)	64 (31.2)	63 (31.2)	
Smoking status, *n* (%)					
Current smoker	283 (46.2)	79 (35.7)	100 (41.8)	104 (68.4)	0.022
Non-smoker	329 (53.8)	126 (57.0)	105 (43.9)	98 (64.5)	
Alcohol consumption, *n* (%)					
Yes	324 (52.9)	108 (48.9)	106 (44.4)	110 (72.4)	0.854
No	288 (47.1)	97 (43.9)	99 (41.4)	92 (60.5)	

PA, physical activity; WHtR, waist-to-height ratio; BMI, body mass index.

### 3.2 Blood pressure control profile

**Figure 1:**
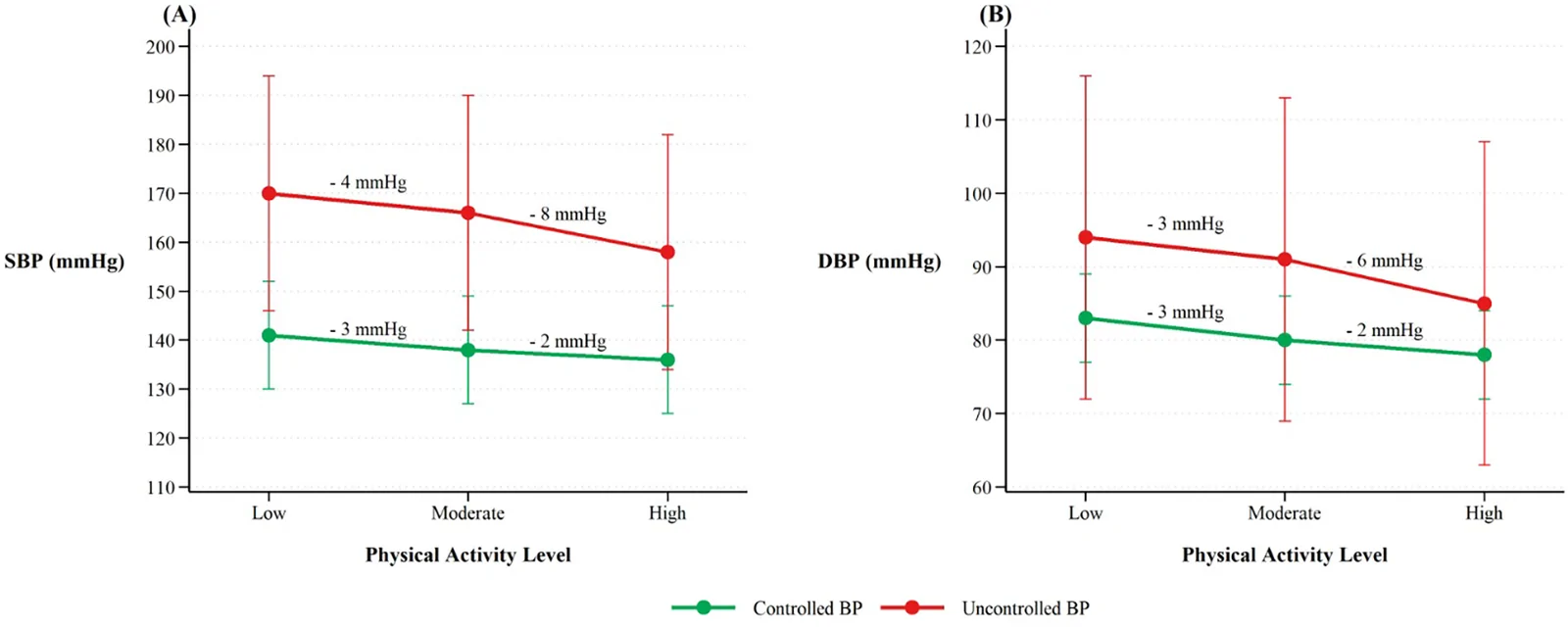
Systolic **(A)** and diastolic **(B)** blood pressure according to physical activity level and blood pressure control status.

Overall, 66.0% of participants had uncontrolled blood pressure, whereas 34.0% achieved the recommended treatment targets. Participants with uncontrolled blood pressure had higher mean systolic and diastolic blood pressure than those with controlled blood pressure (166 ± 24/91 ± 22 mmHg vs. 138 ± 11/80 ± 6 mmHg; both *p* < 0.001). Isolated systolic hypertension was markedly more frequent among participants with uncontrolled blood pressure than among those with controlled blood pressure (68.3% vs. 14.9%; *p* < 0.001). Blood pressure control was observed in 171 of 469 participants receiving monotherapy (36.5%), compared with 37 of 143 participants receiving two or more antihypertensive agents (25.9%; *p* = 0.019). This finding should be interpreted cautiously, as combination therapy is generally prescribed to patients with more severe hypertension. Calcium channel blocker use was more frequent among participants with controlled blood pressure than among those with uncontrolled blood pressure (61.1% vs. 40.8%; *p* < 0.001) (Table 2; Figure 1).

**Table 2:** Blood pressure control among hypertensive patients.

Variables	Overall (*n* = 612)	Controlled BP (*n* = 208)	Uncontrolled BP (*n* = 404)	*p*-value
SBP, mmHg	156 (35)	138 (11)	166 (24)	<0.001
DBP, mmHg	83 (15)	80 (6)	91 (22)	<0.001
Hypertension classification				
Grade 1	45 (7.4)	3 (1.4)	42 (10.4)	<0.001
Grade 2	70 (11.4)	7 (3.4)	63 (15.6)	
Grade 3	26 (4.2)	3 (1.4)	23 (5.7)	
Isolated systolic hypertension	307 (50.2)	31 (14.9)	276 (68.3)	
Antihypertensive medication, *n* (%)				
β-blockers	25 (4.1)	22 (10.6)	3 (0.7)	0.017
Diuretics	63 (10.3)	15 (7.2)	48 (11.9)	0.090
Calcium channel blockers	292 (47.7)	127 (61.1)	165 (40.8)	<0.001
ACE inhibitors	117 (19.1)	54 (26.0)	63 (15.6)	0.002
ARB	115 (18.8)	32 (15.4)	83 (20.5)	0.128
Number of antihypertensive medications, *n* (%)				
Monotherapy	469 (76.6)	171 (82.2)	298 (73.8)	0.019
Two drugs or more	143 (23.4)	37 (17.8)	106 (26.2)	
Time since hypertension diagnosis, *n* (%)				
<5 years	307 (50.2)	111 (53.4)	196 (48.5)	0.256
>5 years	305 (49.8)	97 (46.6)	208 (51.5)	
Medication adherence, *n* (%)				
Good	290 (47.4)	101 (48.6)	189 (46.8)	0.213
Fair	247 (40.4)	82 (39.4)	165 (40.8)	
Poor	75 (12.3)	22 (10.6)	53 (13.1)	
Salt consumption practice, *n* (%)				
High	368 (60.1)	145 (69.7)	223 (55.2)	0.001
Low	244 (39.9)	63 (30.3)	181 (44.8)	

BP, blood pressure; SBP, systolic blood pressure; DBP, diastolic blood pressure; ACE; angiotensin-converting enzyme; ARB, angiotensin receptor blocker.

### 3.3 Factors associated with uncontrolled blood pressure

In univariable analyses, uncontrolled blood pressure was positively associated with age >60 years, family history of hypertension, combined diabetes and dyslipidemia, elevated WHtR, smoking, and high salt intake. In contrast, middle-income status, moderate and high physical activity, and fair medication adherence were inversely associated with uncontrolled blood pressure.

**Figure 2:**
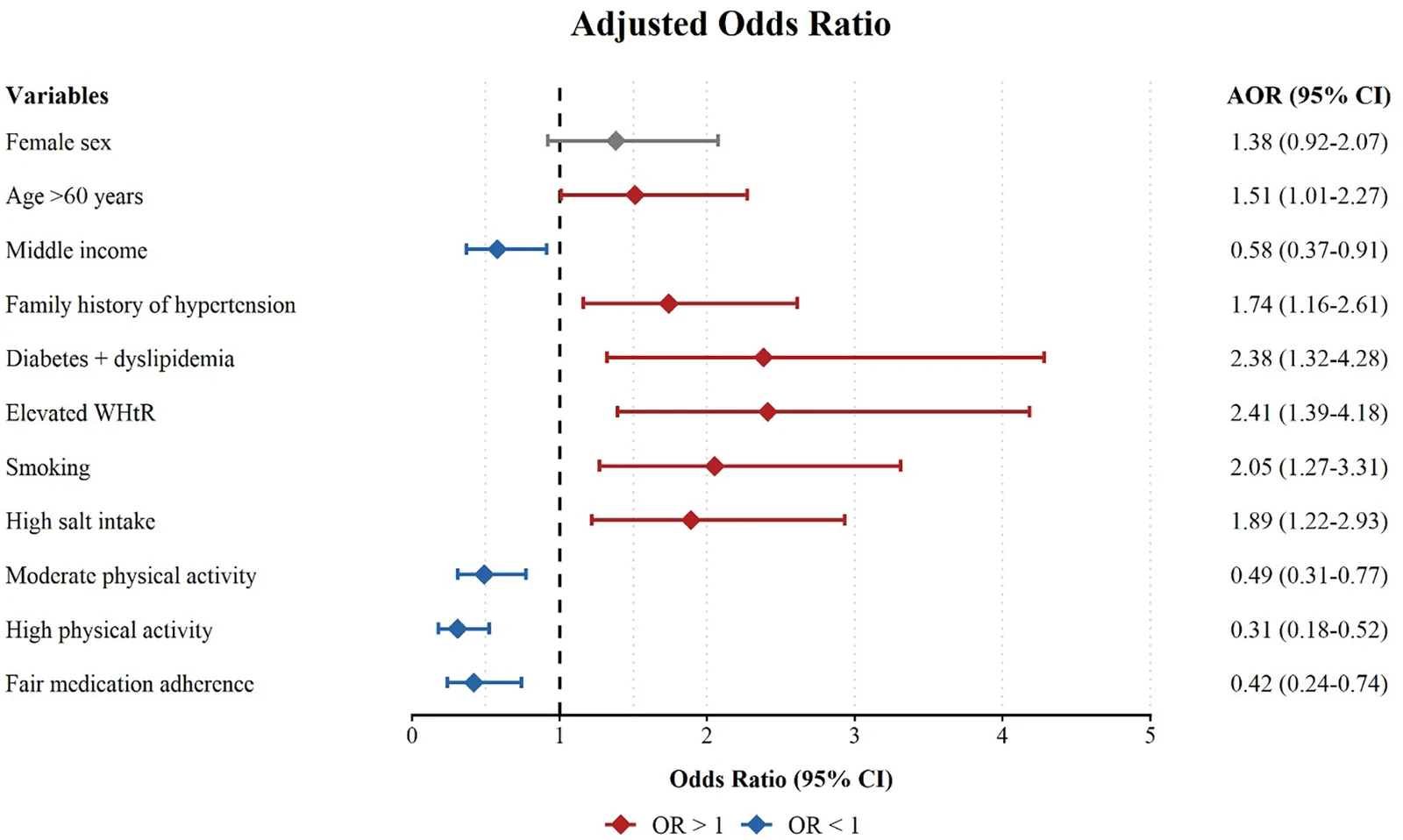
Forest plot of adjusted odds ratios (AORs) for factors associated with uncontrolled blood pressure among hypertensive adults.

In the multivariable logistic regression model, age >60 years (AOR = 1.51; 95% CI: 1.01–2.27; *p* = 0.046), family history of hypertension (AOR = 1.74; 95% CI: 1.16–2.61; *p* = 0.007), combined diabetes and dyslipidemia (AOR = 2.38; 95% CI: 1.32–4.28; *p* = 0.004), elevated WHtR (AOR = 2.41; 95% CI: 1.39–4.18; *p* = 0.002), smoking (AOR = 2.05; 95% CI: 1.27–3.31; *p* = 0.003), and high salt intake (AOR = 1.89; 95% CI: 1.22–2.93; *p* = 0.004) remained independently associated with higher odds of uncontrolled blood pressure. Conversely, middle-income status (AOR = 0.58; 95% CI: 0.37–0.91; *p* = 0.018), moderate physical activity (AOR = 0.49; 95% CI: 0.31–0.77; *p* = 0.002), high physical activity (AOR = 0.31; 95% CI: 0.18–0.52; *p* < 0.001), and fair medication adherence (AOR = 0.42; 95% CI: 0.24–0.74; *p* = 0.003) were independently associated with lower odds of uncontrolled blood pressure, demonstrating a graded inverse association across physical activity levels. No multicollinearity was detected, with a maximum VIF of 3.41. The Hosmer–Lemeshow goodness-of-fit test indicated adequate model calibration (*χ*^2^ = 7.12, *p* = 0.524) (Table 3; Figure 2).

**Table 3:** Factors independently associated with uncontrolled blood pressure among hypertensive adults.

Factor	CORs (95% CI)	*p*-value	AORs (95% CI)	*p*-value
Female sex	1.45 (1.02–2.06)	0.039	1.38 (0.92–2.07)	0.118
Age >60 years	1.72 (1.20–2.45)	0.003	1.51 (1.01–2.27)	0.046
Middle income	0.61 (0.42–0.89)	0.010	0.58 (0.37–0.91)	0.018
Family history of hypertension	1.82 (1.28–2.60)	0.001	1.74 (1.16–2.61)	0.007
Diabetes + dyslipidemia	2.65 (1.58–4.45)	<0.001	2.38 (1.32–4.28)	0.004
Elevated WHtR	2.95 (1.78–4.88)	<0.001	2.41 (1.39–4.18)	0.002
Smoking	2.36 (1.54–3.62)	<0.001	2.05 (1.27–3.31)	0.003
High salt intake	2.12 (1.45–3.11)	<0.001	1.89 (1.22–2.93)	0.004
Moderate physical activity	0.58 (0.39–0.86)	0.007	0.49 (0.31–0.77)	0.002
High physical activity	0.36 (0.23–0.57)	<0.001	0.31 (0.18–0.52)	<0.001
Fair medication adherence	0.51 (0.31–0.83)	0.007	0.42 (0.24–0.74)	0.003

COR, crude odds ratio; AOR, adjusted odds ratio; CI, confidence interval; WHtR, waist-to-height ratio.

## 4 Discussion

This cross-sectional study examined the association between graded physical activity levels and blood pressure control among treated hypertensive adults attending primary healthcare centers in Morocco. Uncontrolled blood pressure was observed in 66.0% of participants. Compared with low physical activity, moderate physical activity was independently associated with 51% lower odds of uncontrolled blood pressure (AOR = 0.49; 95% CI: 0.31–0.77), whereas high physical activity was associated with 69% lower odds (AOR = 0.31; 95% CI: 0.18–0.52), indicating a graded inverse association. The study extends previous Moroccan evidence by evaluating physical activity in relation to standardized blood pressure control while accounting for sociodemographic, behavioral, anthropometric, cardiometabolic, familial, and treatment-related factors. Older age, family history of hypertension, central adiposity, smoking, high salt intake, and combined diabetes and dyslipidemia were independently associated with higher odds of uncontrolled blood pressure.

Recent regional evidence supports our findings. In Marrakech, 73.5% of 922 treated hypertensive patients had uncontrolled blood pressure, with tobacco use (AOR = 4.34), overweight or obesity (AOR = 1.73), and family history of hypertension (AOR = 1.58) identified as independent correlates (19). In the Central African Republic, only 39.5% of 656 patients achieved blood pressure control, and diabetes was associated with a lower likelihood of control (OR = 0.36; 95% CI: 0.25–0.52) (20). Furthermore, a 2025 meta-analysis of eight African trials involving 1,112 treated hypertensive adults found that aerobic activity reduced systolic and diastolic blood pressure by approximately 5.40 and 1.90 mmHg, respectively (21).

The high prevalence of uncontrolled blood pressure indicates that treatment targets are not being achieved for many patients despite antihypertensive therapy. This pattern is consistent with reports from many low- and middle-income countries, where delayed treatment intensification, suboptimal adherence, fragmented follow-up, and limited lifestyle counselling contribute to poor control (13, 19). Similar observations have been reported in African and other resource-constrained settings (22). These results highlight the need for stronger long-term management strategies in Morocco.

A major finding was the graded inverse association between physical activity and uncontrolled blood pressure. Compared with inactive participants, those with moderate and high physical activity had significantly lower adjusted odds of poor control. This is consistent with randomized trials and meta-analyses showing that aerobic, resistance, and combined exercise reduce systolic and diastolic blood pressure (23). Potential mechanisms include improved endothelial function, lower sympathetic activity, enhanced insulin sensitivity, reduced arterial stiffness, and favorable body composition changes. Although causality cannot be inferred, these findings suggest that physical activity may represent a practical and scalable component of hypertension management (24).

Older age was independently associated with poorer blood pressure control, which is biologically plausible given age-related arterial stiffness, endothelial dysfunction, and the higher prevalence of isolated systolic hypertension (25). Family history of hypertension may reflect shared genetic susceptibility and household behavioral factors. Combined diabetes and dyslipidemia were also associated with uncontrolled blood pressure, supporting evidence that metabolic multimorbidity increases treatment complexity and vascular risk (26).

Elevated waist-to-height ratio emerged as an independent correlate of poor control, suggesting that central adiposity may be more informative than general obesity in the Moroccan population (17). Abdominal fat accumulation is linked to sympathetic activation, inflammation, insulin resistance, and renin–angiotensin–aldosterone system dysregulation. Smoking was likewise associated with uncontrolled blood pressure, consistent with the adverse vascular effects of tobacco exposure. High salt intake remained another important determinant, aligning with extensive evidence that sodium excess increases blood pressure, particularly among salt-sensitive individuals (27). Together, these findings indicate that physical activity should be considered within a broader pattern of modifiable behavioral and metabolic factors rather than as an isolated determinant.

Middle-income status and fair medication adherence were associated with lower odds of uncontrolled blood pressure. The association with middle-income status may reflect more consistent access to healthcare, medication continuity, and healthier living conditions. However, socioeconomic status may also capture unmeasured differences in health literacy, food access, and opportunities for physical activity. The inverse association observed for fair adherence should be interpreted cautiously, particularly because adherence was self-reported and may have been affected by reporting or classification bias (28). Further studies using validated adherence instruments and objective medication measures are needed to clarify this finding.

From a practical perspective, physical activity assessment could be incorporated into routine hypertension follow-up in Moroccan primary healthcare centers. Brief individualized counseling, walking-based activities, home-based exercise, and referral to supervised programs where available could complement pharmacological treatment, salt reduction, smoking cessation, weight management, and medication adherence support. Recommendations should be adapted to patients' age, comorbidities, functional capacity, and cardiovascular risk. At the public health level, community-based programs and access to safe, affordable, and culturally acceptable opportunities for physical activity may help reduce barriers to sustained participation. These measures should be regarded as components of comprehensive hypertension care rather than substitutes for appropriate pharmacological treatment.

This study has limitations. Its cross-sectional design precludes causal inference, and reverse causation is possible. Physical activity, salt intake, and adherence were self-reported and may be affected by recall or desirability bias. Residual confounding may also remain despite adjustment for several clinically relevant variables. Finally, recruitment from one public primary healthcare center in Kenitra may limit generalizability to untreated adults, patients followed in other Moroccan regions. Nevertheless, strengths include a relatively large real-world sample, standardized blood pressure measurement, use of a culturally adapted physical activity instrument, and adjustment for multiple clinically relevant covariates.

## 5 Conclusion

This study addressed the persistent challenge of inadequate blood pressure control among treated hypertensive adults by examining whether physical activity levels were associated with hypertension control in a Moroccan primary healthcare population using a cross-sectional analytical design. From a scientific perspective, this study contributes new evidence from North Africa, a region still underrepresented in hypertension lifestyle research. It strengthens current understanding that blood pressure control is shaped not only by pharmacological treatment but also by behavioral and metabolic determinants, with physical activity emerging as a potentially powerful modifiable factor. Practically, these findings are relevant for clinicians, public health planners, and policymakers. Integrating structured physical activity counseling, sodium reduction strategies, smoking cessation support, and waist-to-height ratio screening into primary care services may improve hypertension outcomes at relatively low cost, particularly in resource-constrained settings. Patients may also benefit from clearer lifestyle guidance accompanying medication treatment.

## Data Availability

The datasets used and analyzed during the current study are available from the corresponding author upon reasonable request.
